# Synthesis, Pharmacological Properties, and Potential Molecular Mechanisms of Antitumor Activity of Betulin and Its Derivatives in Gastrointestinal Cancers

**DOI:** 10.3390/pharmaceutics15122768

**Published:** 2023-12-13

**Authors:** Marcel Madej, Joanna Gola, Elwira Chrobak

**Affiliations:** 1 Department of Molecular Biology, Faculty of Pharmaceutical Sciences in Sosnowiec, Medical University of Silesia, 40-055 Katowice, Poland; jgola@sum.edu.pl; 2 Silesia LabMed, Centre for Research and Implementation, Medical University of Silesia, 40-752 Katowice, Poland; 3 Department of Organic Chemistry, Faculty of Pharmaceutical Sciences in Sosnowiec, Medical University of Silesia, 40-055 Katowice, Poland; echrobak@sum.edu.pl

**Keywords:** betulin, derivatives, synthesis, gastrointestinal cancers, colorectal cancer, molecular mechanisms, interleukins

## Abstract

Gastrointestinal (GI) cancers are an increasingly common type of malignancy, caused by the unhealthy lifestyles of people worldwide. Limited methods of treatment have prompted the search for new compounds with antitumor activity, in which betulin (BE) is leading the way. BE as a compound is classified as a pentacyclic triterpene of the lupane type, having three highly reactive moieties in its structure. Its mechanism of action is based on the inhibition of key components of signaling pathways associated with proliferation, migration, interleukins, and others. BE also has a number of biological properties, i.e., anti-inflammatory, hepatoprotective, neuroprotective, as well as antitumor. Due to its poor bioavailability, betulin is subjected to chemical modifications, obtaining derivatives with proven enhanced pharmacological and pharmacokinetic properties as a result. The method of synthesis and substituents significantly influence the effect on cells and GI cancers. Moreover, the cytotoxic effect is highly dependent on the derivative as well as the individual cell line. The aim of this study is to review the methods of synthesis of BE and its derivatives, as well as its pharmacological properties and molecular mechanisms of action in colorectal cancer, hepatocellular carcinoma, gastric cancer, and esophageal cancer neoplasms.

## 1. Introduction

Gastrointestinal (GI) cancers are one of the most common among both men and women worldwide [1]. It is estimated that they account for about a quarter of all cancer types. GIs include esophageal cancer (EC), gastric cancer (GC), hepatocellular carcinoma (HCC), and colorectal cancer (CRC), among others [1]. In most cases, inappropriate lifestyles associated mainly with the consumption of large amounts of highly processed meat, smoking, excessive alcohol intake, maintaining a low-quality diet, and low or lack of physical activity are responsible for their development [1,2]. For GC, an increased risk is also observed in those infected with *Helicobacter pylori* and those suffering from reflux disease [3]. In contrast, for HCC, hepatitis B or hepatitis C virus infection also has a significant impact on morbidity [1,2,3]. In addition, people with inherited genetic mutations directly linked to the development of cancer, such as mutations in the *APC* gene or the *BRAF* gene, have a higher risk of developing GI cancer [1,2,3].

Current GI cancer therapy mainly involves the use of a broad group of chemotherapeutics, i.e., 5-fluorouracil (5-FU), cisplatin, or its combination with radiotherapy [3,4]. For solid tumors, surgical resection of the cancerous lesion with a margin of healthy tissue is also performed [3,4]. However, this does not always lead to a complete cure for the patient due to the recurrence of the cancer. Despite the relatively good efficacy of chemotherapeutics, they also show high toxicity to the patient’s normal cells [3]. Therefore, modern medicine and pharmacy are looking for new therapeutic solutions that are free from affecting healthy cells and unaffected by cancerous lesions. One of the more widely researched sources of new compounds with anticancer effects are substances of natural origin with proven biological activity [5,6]. Among them, major research has focused on curcumin, fisetin, kaempferol, galangin, resveratrol, and betulin (BE) [5,7]. Substances isolated from natural raw materials, mainly plants, are leading the way in the search for new compounds with potential anticancer, anti-inflammatory, antiviral, and antimicrobial activities [5,6].

**Figure 1:**
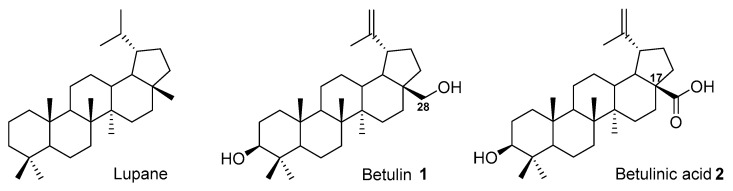
Chemical structure of lupane, betulin **1**, and betulinic acid **2**.

BE, known chemically as lup-20(29)-ene-3β,28-diol, is classified as a lupane-type pentacyclic triterpene [5]. The most important derivatives of betulin **1** include betulinic acid (BA) **2**, a product of the oxidation of the primary hydroxyl group at C-28 [5]. This compound contains a carboxyl function at the C-17 position, which allows it to be transformed into various derivatives different from those obtained from BE [5] (Figure 1).

BE is a compound of natural origin that is present in the largest amount in the outer layer of the bark of trees of the Betula species (*Betula alba*, *Betula pubescens*, *Betula verrucosa*, *Betula pendula*, and *Betula papyrifera*) [8]. Research on the composition of extracts from other plants allowed us to expand the group of potential sources of this compound [8]. The presence of BE has been found, among others, in the bark of plants from the families *Platanaceae*, *Adoxaaceae*, *Oleaceae*, *Rhamnaceae*, *Fagaceae*, and *Fabaceae*, as well as in other parts of plants from various families, e.g., leaves (*Apocynaceae*, *Amaranthaceae*, and *Ebenaceae*), roots (*Ranunculaceae* and *Iridaceae*), fruits (*Rosaceae*), and aerial parts of plants (*Asteraceae*, *Acanthaceae*, and *Polygonaceae*) [8].

Substances with a triterpene structure are most often obtained via extraction from crushed birch bark using various organic solvents or their mixtures. The extracts obtained are recrystallized or purified using chromatographic methods [9,10,11]. BE of good purity can be obtained with high efficiency using ionic liquids [12]. Armbruster et al. [13] described the extraction of birch bark using supercritical fluid technology [13]. Another way to extract BE is through thermal sublimation [14].

This review article is so far the first comprehensive compilation of all the essential information on BE derivatives in gastrointestinal cancers, demonstrating further possibilities for the use of the most promising derivatives in future studies in both drug formulation technology and in vivo model studies. Therefore, the aim of this paper is to review recent reports on the synthesis, pharmacological properties, and potential molecular mechanisms of the antitumor activity of BE and its synthetic derivatives against CRC cells, HCC, GC, and EC.

## 2. Properties and Molecular Mechanisms of Betulin and Its Derivatives against Cancer Diseases

### 2.1. Pharmacological Properties of Betulin and Its Derivatives

An integral part of the process of carcinogenesis in the development of gastrointestinal tract tumors is the occurrence of chronic inflammation [15,16]. During its initial phase, an influx and activation of immune cells occurs, i.e., macrophages, dendritic cells, and neutrophils, which constitute the first line of defense against the disease process [15,16]. As a result of a cascade of cytokines secreted by such cells, i.e., interleukins (ILs), interferons (INFs), or growth factors, they trigger the appearance of the state of inflammation [15,16]. It is also assumed that nitric oxide (NO), generated from l-arginine and influenced by the enzyme inducible nitric oxide synthase (iNOS), is a key factor in the development of chronic inflammation, while cyclooxygenase 2 (COX-2) is responsible for the development of inflammatory symptoms [15,16]. In studies, it has been proven that both BE and BA are able to reduce both NO and COX-2 activities, as well as play a crucial role in decreasing the production of pro-inflammatory interleukins, e.g., IL-1β, IL-6, or IL-8 [15,16].

BE has also been shown to exhibit antiviral and antimicrobial activity [17,18]. Studies by other authors have confirmed the effect of betulin on viruses such as influenza FPV (H7N1), SARS-CoV-2 Mpro, dengue virus (DENV-NS5 RdRp), herpes simplex virus types I and II (HSV-1 and HSV-2), enteric cytopathic human orphan virus 6 (ECHO 6), human cytomegalovirus (HCMV), vesicular stomatitis virus (VSV), and encephalomyocarditis virus (EMCV) [19,20,21,22,23,24]. In the first case, studies conducted on the effects of BE and its derivatives on human immunodeficiency virus type-1 (HIV-1) infection demonstrate that it interferes with viral maturation and entry into the host cell [17]. Studies by Bębenek et al. [18] also show that acetylenic and triazole derivatives exhibit predictive activity against vesicular stomatitis virus (VSV) and herpes simplex virus type-1 (HSV-1). Thus, this shows that compounds of naturalized origin and their appropriate modifications can provide a large source of potent antiviral drugs [17,18]. BE and its derivatives also display antibacterial activity against bacteria, i.e., *Bacillus subtilis* or *Staphylococcus aureus*, and have proven bacteriostatic activity against *Klebsiella pneumoniae* [17,18].

Furthermore, BE, as well as its derivatives, also exhibit antitumor activity, which will exhibit a different mechanism of action depending on the type of tumor [5,17]. Reports indicate that its mechanism of action affects signaling pathways directly related to the proliferation of neoplastic cells, cell death via apoptosis, or pathways involved in the angiogenesis process [5,17]. The main potential mechanisms include the following: influence on interleukin-related pathways, nuclear factor kappa B (NF-kB), nuclear factor erythroid 2-related factor 2 (Nrf2), inhibition of reactive oxygen species (ROS), and mitogen-activated protein kinases (MAPK) pathways [5,17].

**Figure 2:**
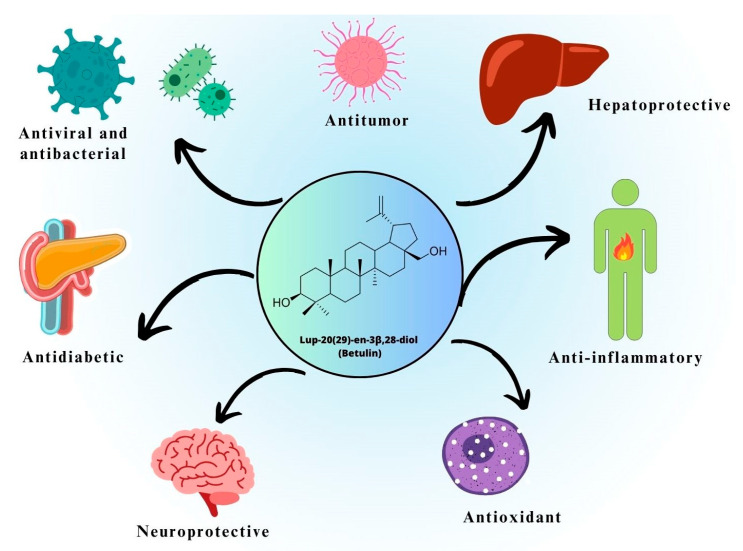
Potential pharmacological properties of betulin and its derivatives.

Additionally, the triterpenes have proven neuroprotective, hepatoprotective, antidiabetic, and antioxidant effects [25,26,27]. The major pharmacological properties of BE and its derivatives are summarized in Figure 2.

### 2.2. Effects on Interleukin-Related Pathways

Oxidative stress, bacterial toxins, or genotoxic agents are among the main causes of “reprogramming” the physiological action of interleukins into a pathological process, resulting in tumor growth, progression, and the possibility of metastasis [28,29,30]. Persistent chronic inflammation in cancers, in response to the aforementioned factors, results in the activation of the transcription factor NF-kB, thereby stimulating the production of an inactive form of IL-1β, or pro-IL-1β, and the NLR family pyrin domain containing 3 protein (NLRP3) inflammasome [28,29,30]. Fibroblasts, epithelial cells, and dendritic cells, i.e., macrophages and monocytes, are among the first cells to respond to the appearance of these two proteins by releasing functional cytokines: IL-1β, IL-6, and IL-11 [28,29,31]. In addition, the presence of IL-33, derived from tumor-initiating cells, results in the recruitment of macrophages that secrete TGFβ, which inactivates cytotoxic T cells, preventing them from properly neutralizing pathogens, including cancer cells [28,29]. As IL-1β concentrations increase, progressive damage to genetic material occurs due to the increased production of ROS and NO via epithelial cells [28,29]. The presence of IL-6, IL-11, and IL-12 under pathological conditions enhances the phosphorylation response of the transcription factor signal transducer and activator of transcription 3 (STAT3), contributing to excessive proliferation of tumor cells as well as inducing the epithelial–mesenchymal transition (EMT) [28,29]. The cascade of all events is further looped through the induction of IL-17 production, a natural activator of NF-kB [28,29]. A significant influence on the progression of cancer is also exerted by the cells that form the so-called tumor microenvironment (TME). This structure includes not only immune cells but also cancer-associated fibroblasts (CAFs) or tumor-associated endothelial cells (TECs) [28]. In addition, receptors play an important role in the process of activating immune cells to secrete the appropriate interleukins, which allow the protein to interact with the target cell, contributing to a specific effect [28,32]. Compounds of natural origin, mainly betulin, affect the pathway related to cyclooxygenases (COX) and also the transcription factor NF-kB [33,34]

Due to the fact that betulin exhibits anti-inflammatory effects, research was performed to evaluate its effects on the production of pro-inflammatory cytokines [32,35,36]. Zhao et al. [36] conducted the investigation on a mouse model of induced sepsis. In the test, the scientists administered 4 mg/kg or 8 mg/kg of betulin intraperitoneally to rats, depending on the study group [36]. After analysis, they demonstrated a significant decrease in the expression of the major pro-inflammatory interleukins, i.e., IL-6 and IL-1β, as well as TNF-α [32,36]. In addition, they concluded that the decrease may be due to inhibition of the NF-kB signaling pathway and MAPK [36].

In order to improve pharmacokinetic and pharmacological properties, betulin undergoes chemical modifications to obtain more functional derivatives [37,38]. Studies conducted by Lubczyńska et al. [38] have shown, using oligonucleotide microarrays, that alkynyl derivatives of betulin affect the expression profile of genes closely related to the inflammatory status of colorectal cancer [38]. This suggests, therefore, that betulin itself, as well as its synthetic derivatives, may have a significant impact on the inflammatory response in tumors by regulating key cytokines and inflammatory mediators that promote tumor development.

### 2.3. Activation of Nrf2 Pathway

Significant roles in this process are played by the two transcription factors, Nrf2 and NF-kB, whose main functions are to maintain normal homeostasis in the body [39,40]. In the first case, Nrf2 tightly regulates the expression of antioxidant proteins, mainly phase II cytoprotective enzymes, i.e., heme oxidase-1 (HO-1), superoxide dismutase (SOD), etc., leading to protection of the cell against ROS-related damage, which is produced in large amounts by pathogens such as bacteria or neoplastic cells [40,41]. Nrf2 can be regulated both at the transcriptional level by NF-kB, aryl hydrocarbon receptor (AhR), or activating transcription factor 4 (ATF4), as well as post-transcriptionally or post-translationally [42,43]. The process of Nrf2 factor phosphorylation is triggered by keap1, MAPK, and PI3K/Akt, resulting in Nrf2 translocation [35,43].

The mechanism of action of BE is suggested to be via increased expression of HO-1 and Nrf2 and simultaneous inhibition of AhR receptor and keap1 protein expression [35,43,44]. It has also been suggested that BE enhances the expression of enzymes involved in glutathione metabolism, i.e., glutathione S-transferase (GST) [35,44,45]. The concomitant action of antioxidant enzymes also reduces the production of ROS, which further protects normal cells from damage. Decreased HO-1 levels result in the biochemical conversion of heme, producing an anti-inflammatory effect and releasing cytokines essential to the proper functioning of the immune system in the defense against cancer [35,46,47,48].

Interestingly, studies by Huang et al. [49] show that inhibition, in contrast, of Nrf2 activity in colorectal cancer results in cell sensitivity to chemotherapeutic agents by inducing ferroptosis and pyroptosis in cells [35,49]. This suggests, therefore, that the potential molecular mechanism of action of BE is based on a temporary activation of Nrf2, resulting in inhibition of neoplastic cell proliferation, but it should be noted that long-term activation of Nrf2 may also have a beneficial effect on tumor cells [35,49].

**Figure 3:**
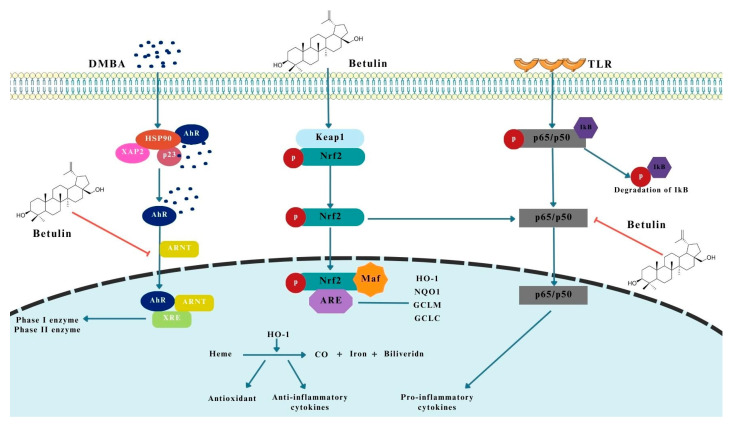
Molecular effect of betulin on Nrf2 transcription factor in gastrointestinal cancers [35]. The figure was partly generated using Servier Medical Art, provided by Servier and licensed under a Creative Commons Attribution 3.0 unported license.

The molecular mechanisms of betulin activity on the transcription factor Nrf2 are shown in Figure 3.

### 2.4. Activation of NF-kB Pathway

The other transcription factor, NF-kB, is responsible for keeping the immune system functioning correctly in response to stress factors such as autoimmune diseases, viral infections, or neoplastic cells [35,46,47]. Several factors influence the activation process of NF-kB. However, the most important are tumor necrosis factor (TNF), proinflammatory interleukins, bacterial lipopolysaccharide (LPS), or ultraviolet radiation (UV) [46,47].

Primarily, its activity is thought to modulate the secretion of cytokines, enzymes, or growth factors. It is well known that a decrease in NF-kB levels is closely correlated with apoptosis-related cell death, while an increase in NF-kB levels enhances processes associated with the expression of antiapoptotic genes or anti-inflammatory cytokines [35,46,47]. The transcription factor has its own specific inhibitor (IkBα), which is crucial in the regulation of transcription factor expression [35,48,49].

The molecular mechanism of action of BE is suggested to be the down-regulation of phosphorylated NF-kB and its inhibitor, leading to the protection of normal cells [35,48]. In a study conducted by Wang et al. [50] involving mice, BE inhibited the expression of IL-6 and TNFα while increasing the expression of IL-10, which exhibits anti-inflammatory properties [50]. The researchers concluded that this effect was due to inhibition of NF-kB and IkBα phosphorylation and disturbance in NF-kB translocation [35,50].

**Figure 4:**
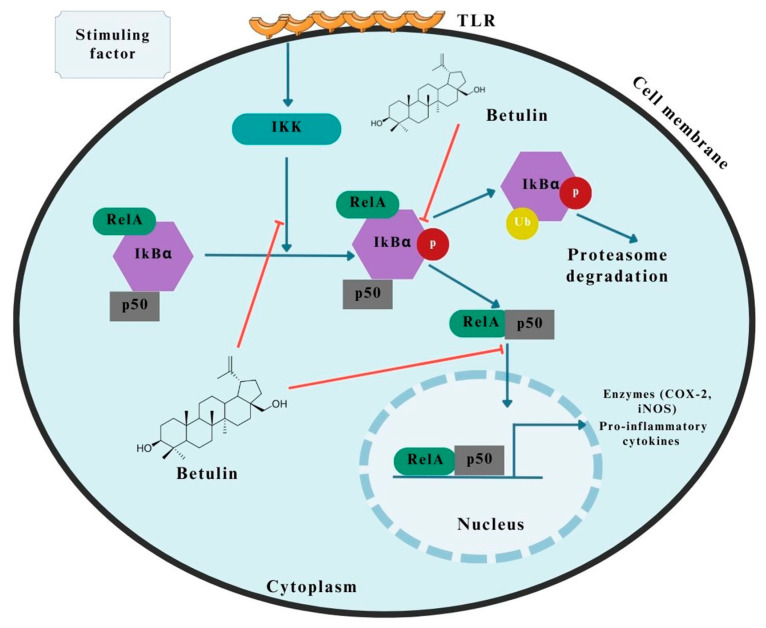
Molecular effect of betulin on NF-kB transcription factor in gastrointestinal cancers [35]. The figure was partly generated using Servier Medical Art, provided by Servier and licensed under a Creative Commons Attribution 3.0 unported license.

Therefore, this suggests that BE affects inflammation in the NF-kB-related pathway, causing inhibition of pro-inflammatory cytokine secretion in favor of enhancement of anti-inflammatory cytokine expression, which may also be reflected in gastrointestinal cancers, one of the key features of which is the occurrence of chronic inflammation [35,50]. The potential molecular mechanism of betulin’s action on the NF-kB pathway is shown in Figure 4.

### 2.5. Induction of Apoptosis Associated with MAPK Pathway Activation

The MAPK pathway is one of the key signaling pathways in tumor progression. It is responsible for modulating proliferation, apoptosis, inflammation, and cell division [35,51]. MAPKs are categorized as protein kinases that exhibit phosphorylation properties. The process of MAPK kinase activation occurs through the stimulation of its three protein kinases, MAP3K, MAP2K, and MAPK, in turn [35,52]. MAP3K is activated by interacting with small GTPases or with the assistance of receptors on the cell surface. Activated MAP3K subsequently phosphorylates MAP2K, which in turn continues to MAPK [35,52]. In this form, the protein kinase is capable of phosphorylating multiple factors in both the cytoplasm and cell nucleus, indirectly leading to changes in gene expression [35,53].

**Figure 5:**
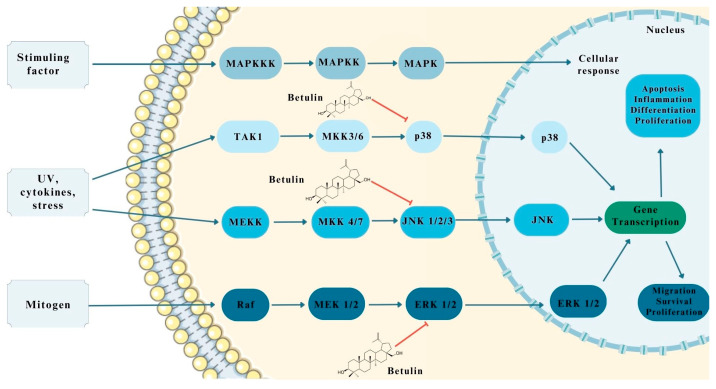
Potential effect of betulin on MAPK pathway in gastrointestinal cancers. The figure was partly generated using Servier Medical Art, provided by Servier and licensed under a Creative Commons Attribution 3.0 unported license.

Another three proteins belong to the MAPK family, i.e., extracellular signal-regulated kinases-1 (ERK-1), p28, and c-jun *N*-terminal kinases (JNK), which provide a trigger point for increasing the free radical pool [35]. In studies on the molecular mechanism of betulin’s action on the MAPK pathway, it has been suggested that BE has an inhibitory effect on MAPK proteins [35,54,55,56]. Research on betulin in colorectal cancer has proposed that it prevents the hyperphosphorylation of ERK, JNK, and p38 in response to stress factors [55,56]. Based on this information, it was concluded that the action of BE is mainly based on directing MAPK protein signaling, thereby reducing inflammation, inhibiting neoplastic cell proliferation, and inhibiting the recurrence of neoplasms [35,56]. Because this study was conducted on CRC, it is likely that the same effect would also be observed in other gastrointestinal cancers; however, more research is needed [35]. A schematic illustration of the mechanism of action of betulin on MAPK is provided in Figure 5, which was created on the basis of Adepoju et al. [35].

### 2.6. Inhibition of ROS Production in Cancer Cells

Gastrointestinal-related cancers exhibit an increased production of ROS than in the case of body homeostasis. High levels of ROS cause DNA damage, inactivate tumor suppressor genes, and act as signaling molecules, interacting directly with receptors to disrupt signaling pathways [35,57,58]. ROS also leads to chronic inflammation by activating COX-2 secretion, pro-inflammatory interleukins, and even TNFα [35,41,59]. Therefore, in order to protect themselves from the adverse effects of ROS, cells possess antioxidant systems, which include enzymes such as glutathione reductase, catalase, and transmembrane dismutase, leading to a decrease in ROS in the cell [35,59,60]. Studies have shown that BE reduced superoxide production in hepatocellular carcinoma cells and also reduced the amount of hydrogen peroxide generated via ROS [61,62]. In addition, BE has also been shown to exhibit antioxidant properties by scavenging free hydroxyl radicals. Interestingly, in a mouse model, BE was found to reduce proinflammatory cytokine levels [35]. This suggests that betulin may also have a similar effect on other gastrointestinal tract-related novelties that involve the production of large amounts of reactive oxygen species [35].

However, the molecular mechanisms of action of BE have not yet been extensively studied. More research is being carried out using the oxidized form of betulin; however, this does not change the fact that betulin itself has promising properties for further research, as do its derivatives.

## 3. Synthesis of Betulin Derivatives 

The betulin skeleton became the basis for the design of numerous derivatives whose structure combines a pentacyclic triterpene system with a known biological effect with other pharmacophore groups. Triterpenes are a group of structurally diverse compounds characterized by the presence of about 30 carbon atoms. Due to the number of rings in their structure, there are two main groups of triterpenes. The first of them are tetracyclic triterpenoids; the second large group consists of pentacyclic triterpenoids. Due to their structure, pentacyclic triterpenoids have been divided into six types: fridelan, lupan, ursan, olean, serratan, and taraxastan [63]. Particular attention was paid to the study of the pharmacological activity of compounds with structures such as lupane, olean, and ursan. The biological activity of these compounds covers a diverse range of activities, e.g., anti-inflammatory, hepatoprotective, cardioprotective, antibacterial, antiviral, antioxidant, antihypertensive, antiulcer, and anticancer [64]. There are numerous literature reports devoted to the study of anticancer activity, including the effect of compounds with this structure against human hepatocellular carcinoma, gastric adenocarcinoma, and colorectal cancer cells [64,65,66,67]. 

**Figure 6:**
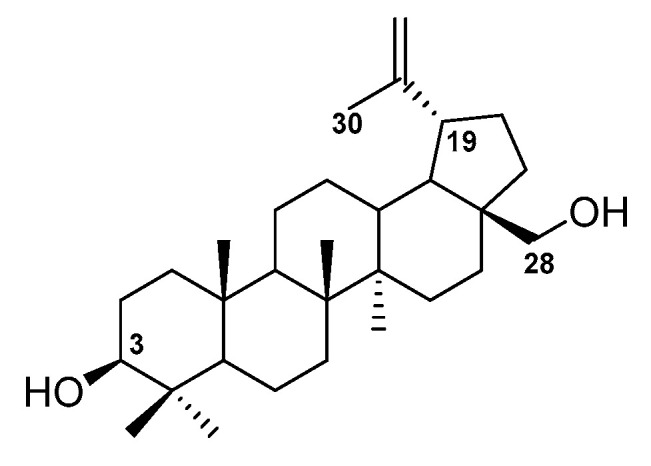
The most chemically reactive centers in structure of betulin **1**.

The introduction of new functional groups into the triterpene scaffold provides an opportunity to obtain new compounds with higher activity, better selectivity, and more favorable physicochemical and pharmacokinetic parameters. The BE molecule contains two chemically active centers, which are hydroxyl groups in positions 3 and 28. The most common way to modify this compound is to use the reactivity of these substituents and transform them into ester, ether, glycosidic, or amide groups. Another potentially reactive site in the BE structure is the isopropenyl substituent at position 19 and the allylic carbon atom C-30 in it (Figure 6).

**Figure 7:**
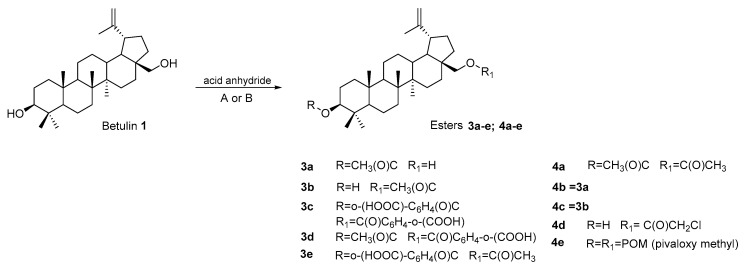
Scheme for the synthesis of ester derivatives of betulin **3a–e** (for **3c–e**, procedure A: DMAP, pyridine, and boiling temperature [68]) and **4a–e** (for **4d,e**, procedure B: CH_2_Cl_2_, full microwave power, and 90 °C under a maximum pressure of 10 bar) [70].

Among the BE derivatives synthesized and tested as potential anticancer agents targeting various types of CRC cell lines, esters are the most numerous. The transformation of the hydroxyl group into the ester function can be performed using appropriate acylating agents. Acid anhydrides are often used for this purpose [68,69,70]. The reaction can be performed using an appropriate anhydride in a solution of pyridine with a catalyst (N,N-dimethylaminopyridine—DMAP) and carried out at the boiling temperature of the reaction mixture (compounds **3a–e**, procedure A). Using acetic anhydride, 3- and 28-acetylbetulin (**3a** and **3b**) were obtained, while in the reaction, phthalic anhydride derivatives **3c–d** were formed (Figure 7). In the reaction with methyl or ethyl substituted phthalic anhydride, compounds were obtained that were devoid of cytotoxic activity towards human colorectal adenocarcinoma (HT-29) [68]. The phthalic derivatives **3c–d** showed higher cytotoxicity (IC_50_ equal to 129.1; 13.6; and 36.1 μM, respectively) towards HT29 cells compared to inactive betulin and monoacetyl derivatives **3a** and **3b** (IC_50_ equal to 154.3 and 76.7 μM) [68].

The team of Kommera et al. used a microwave reactor to react BE with acetic anhydride, chloroacetic anhydride, lauryl, and pivaloxymethyl, conducting the reaction in a dichloromethane solution (Figure 7: compounds **4a–e**, procedure B). The obtained monoester derivatives (**4a–d**) showed good or moderate activity against colorectal (IC_50_ values in range 10.96–41.66 μM for HT-29 and 4.59–87.19 μM for DLD-1) and colon carcinoma cell lines (IC_50_ values in range 12.58–54.06 μM for HCT-8; 4.07–34.94 μM for HCT-116; and 7.42–47.01 μM for SW480). In a study on human fibroblast cells WWO70327, the 4d derivative was less toxic, and the calculated selectivity index (SI) for the DLD-1, HCT-8, HCT-116, and SW 480 cell lines was 3.45, 1.26, 3.89, and 2.13, respectively. The 3,28-Dipivaloxybetulin (**4e**) was the least active, while the product, formed in the reaction with lauryl anhydride with an extended carbon chain (C(O)(CH_2_)_9_CH_3_), turned out to be completely inactive against the tested cell lines [69,70].

**Figure 8:**
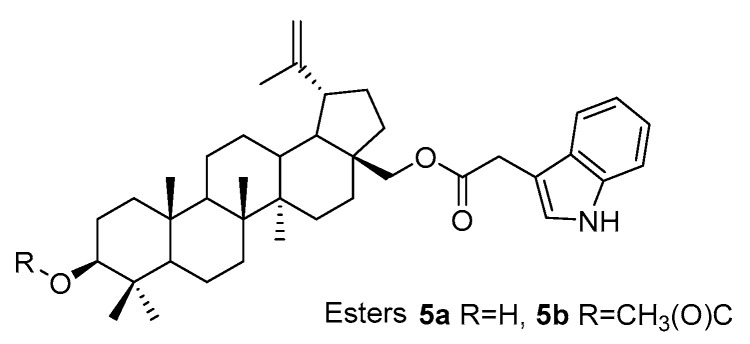
Structure of indolyl derivatives of betulin and 3-acetylbetulin (**5a** and **5b**).

In the acylation of betulin **1** and 3-acetyl betulin **3a** with indolyl acetic acid, derivatives were formed, combining two pharmacophoric fragments in their structure: the betulin skeleton and the heterocyclic indole system, compounds **5a** and **5b**, respectively (Figure 8) [71].

The synthesis was carried out at room temperature with the Steglich method using *N*,*N′*-dicyclohexylcarbodiimide (DCC) and 4-dimethylaminopyridine (DMAP). Anhydrous dichloromethane was used as the solvent. The cytotoxic activity of the obtained derivatives **5a** and **5b** was tested on human colorectal adenocarcinoma cells HT-29 and DLD-1 [71].

**Figure 9:**
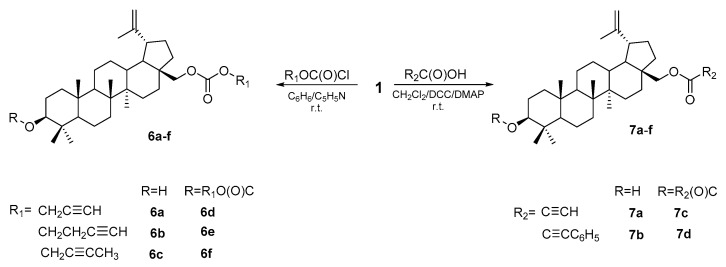
Structure of alkynyl esters of betulin **6a–f** and **7a–d** [72].

The same procedure was used to introduce an ester substituent containing a carbon-carbon triple bond into the betulin molecule, obtaining 28-monosubstituted (**7a,b**) and 3,28-disubstituted derivatives (**7c,d**). In the reaction of BE with chloroformates in a benzene solution and in the presence of pyridine, appropriate mono (**6a–c**) and disubstituted (**6d–f**) derivatives were formed (Figure 9). 

The assessment of the effect of BE and synthesized esters on the cells of the SW-707 line showed that compounds **6a** and **7c** were the most active (IC_50_ = 9.2 and 9.5 μM, respectively; for BE, IC_50_ = 22.9 μM), but their effect was weaker than that of 28-acetylbetulin **3b** (IC_50_ = 5.1 μM) [72]. 

**Figure 10:**
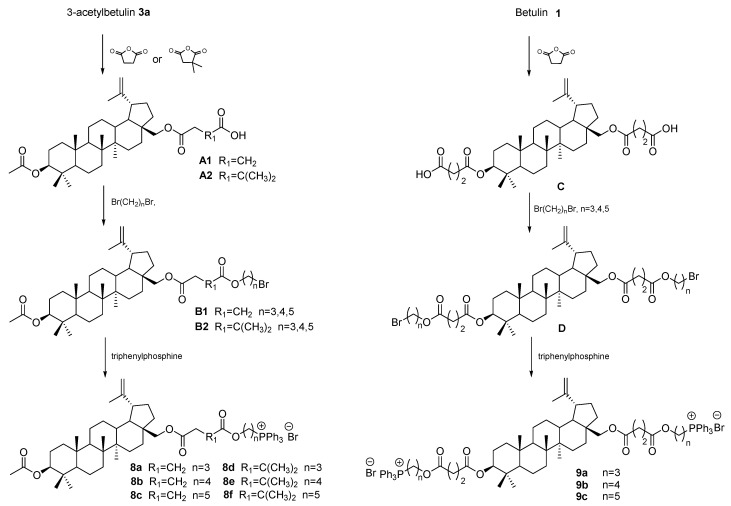
Scheme of the synthesis of triphenylphosphonium derivatives **8a–f** and **9a–c** [73].

Obtaining BE derivatives containing an ester group may also be the first step enabling further functionalization. The reaction of betulin **1** and 3-acetylbetulin **3b** with dicarboxylic acid anhydrides (dimethylsuccinic and succinic) in pyridine in the presence of DMAP at boiling temperature leads to the formation of the corresponding mono (**A1** and **A2**) and dicarboxyacyl derivatives **C** (Figure 10). The presence of the terminal carboxyl group enables the reaction with dibromoalkanes of different carbon chain lengths (*n* = 3–5), which takes place in a mixture of dimethylformamide with acetonitrile, resulting in the formation of 3-O-acetyl-28-O′-(3′-(bromoalkoxycarbonyl)propanoyl)betulin (**B1**), 3-O-acetyl-28-O′-(3′,3′-dimethyl-3′-(bromoalkoxycarbonyl)propanoyl)betulin (**B2**), and 3,28-di(3′-(bromoalkoxycarbonyl)propanoyl)betulin (**D**). In the final phase of the synthetic pathway described by Grymel et al. [73], reactions of compounds **B1**, **B2**, and **D** with triphenylphosphine were carried out in an argon atmosphere without a solvent to obtain betulin hybrids with an alkyltriphenylphosphonium moiety, the structure of which facilitates penetration through biological membranes, including the mitochondrial membrane. The obtained 28-alkyltriphenylphosphonium derivatives of 3-acetylbetulin **8a–f** and 3,28-bisalkyltriphenylphosphonium betulin **9a–c** containing a lipophilic cation are known for their better solubility, increased cytotoxicity, and selectivity of action on cancer cells. In a study on the HCT-116 cell line, derivatives **8a–c** containing a linker without CH_3_ substituents were characterized by higher cytotoxic activity (IC_50_ values equal 5.56, 5.77, and 6.48 μM, respectively) than derivatives containing these groups, among which compound **8f** was the most active (IC_50_ = 12.71 μM). The 3,28-disubstituted compounds **9a–c** were characterized by slightly lower IC_50_ values (6.32, 7.97, and 18.99 μM, respectively). Betulin **1** was inactive in this study [73].

BE conjugates containing sugar fragments were also assessed for cytotoxic activity against colorectal cancer cells [74,75,76,77]. Natural products with a saponin structure are considered to have better pharmacokinetic properties than their aglycones. The sugar groups present in them have a beneficial effect on processes such as absorption, distribution, metabolism, and elimination of the drug molecule. These compounds are more soluble in water.

**Figure 11:**
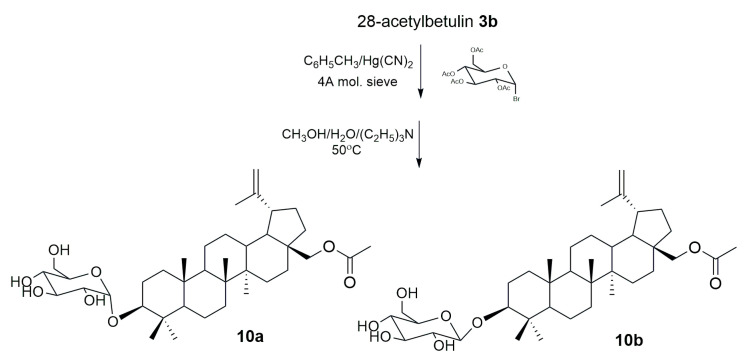
Synthesis of two anomeric glycosides of 28-O-acetylbetulin **10a** and **10b** [74].

Kommera et al. [74] performed a reaction of 28-acetylbetulin **3b** with 2′,3′,4′,6′-tetra-*O*-acetyl-α-D-glucopyranosylbromide in dry toluene with 4Å molecular sieves in the presence of mercury cyanide. Two anomers of the acetylglycoside derivative of compound **3b** were isolated from the resulting mixture, which was then hydrolyzed in an alkaline medium (MeOH/H_2_O/Et_3_N) to obtain 28-*O*-acetylbetulin-3-yl-β-D-glucopyranoside (10a) and 28-*O*-acetylbetulin-3-yl-α-D-glucopyranoside (**10b**) (Figure 11). The study of the cytotoxic activity of the target compounds against cells of the HT-29, DLD-1, HCT-8, HCT-116, and SW480 lines did not show a significant difference in the activity of the two anomers (for **10a** IC_50_ = 4.45; 10.19; 7.46; 9.80; and 9.52 μM and for **10b** IC_50_ = 5.44; 9.86; 5.26; 10.08; and 9.61 μM, respectively) [74].

**Figure 12:**
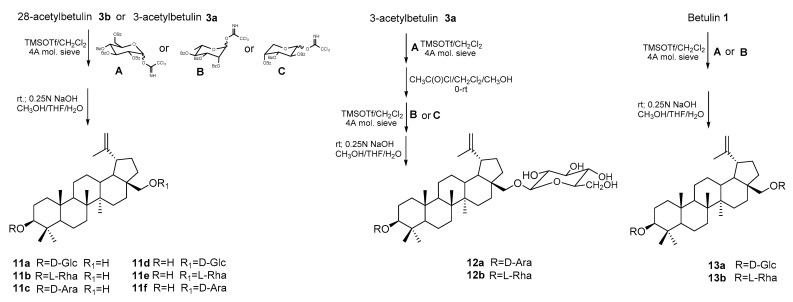
Synthesis of betulin glycosides **11a–f** [75] and bidesmosidic betulin saponins **12a**, **12b**, **13a**, and **13b** [76].

Gauthier et al. [75] carried out the glycosidation of 3- or 28-acetylbetulin (**3a**; **3b**) using specially prepared reagents with activated sugar, which were trichloroacetimidate derivatives of 2,3,4,6-tetra-*O*-benzoyl-α,β-D-glucopyranose (**A**), 2,3,4,6-tetra-*O*-benzoyl-α,β-***L***-rhamnose (**B**), and 2,3,4,6-tetra-*O*-benzoyl-α,β-D-arabinose (**C**). The reaction was carried out at room temperature in a dichloromethane solution in the presence of catalytic amounts of Lewis acid (TMSOTf-trimethylsilyltrifluoromethane sulfonate) (Figure 12). The synthesized betulin glycoside monoderivatives **11a–f** showed low cytotoxic activity (IC_50_ values above 30 μM) towards the DLD-1 line compared to unsubstituted betulin (IC_50_ = 6.6 μM) [75]. 

Researchers from the same team developed the synthesis of bidesmosidic betulin saponins (Figure 12). In the glycosydation reaction of 3-acetylbetulin **3a** with 2,3,4,6-tetra-*O*-benzoyl-D-glucopyranosyl trichloroacetimidate (**A**), a 28-glycosidic derivative was obtained. This product was deacetylated at the C-3 position by reaction with acetyl chloride (CH_3_C(O)Cl) in dry dichloromethane with methanol (1:2; *v*/*v*) and then coupled with 2,3,4-tri-***O***-benzoyl-α-L-arabinopyranosyl trichloroacetimidate (**C**) or 2,3,4-tri-*O*-benzoyl-α-L-rhamnopyranosyl trichloroacetimidate (**B**). In the last step, benzoylated bidesmosides were deprotected via hydrolysis under alkaline conditions (NaOH, MeOH/THF/H_2_O) to obtain the target compounds **12a,b** [76]. 

The authors glycosidated unprotected BE at both the C-3 and C-28 positions. For this purpose, they used the reverse Schmidt procedure. Betulin **1** and the catalyst (TMSOTf) were pre-mixed, and excess sugar (**A** or **B**) was added dropwise at a low temperature (−10 °C). Benzyl groups of sugar fragments are hydrolyzed in an alkaline medium. Also, most bidesmosidic saponins were characterized by lower activity against the DLD-1 line (IC_50_ in the range 10.6–27 μM) than betulin (IC_50_ = 6.6 μM). The exception was the rhamnopyranose derivative **13b**, for which the highest activity was determined (IC_50_ = 1.9 μM) [76]. 

**Figure 13:**
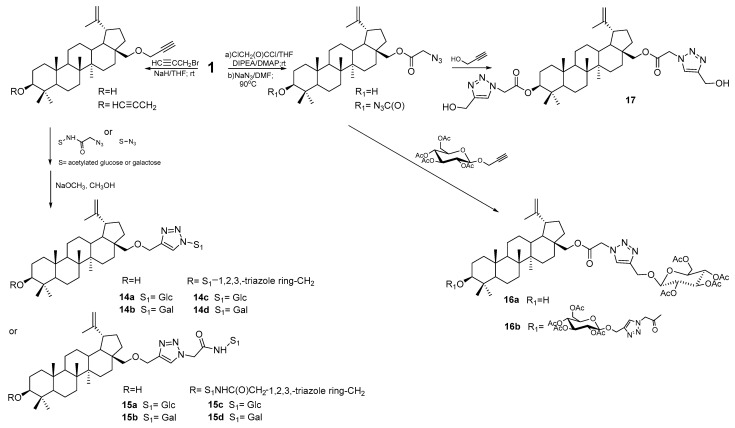
Synthesis of betulin glycoconjugates **14a–d**, **15a–d**, **16a,b**, and **17** [77].

Grymel et al. [77] synthesized a series of new BE glycoconjugates (Figure 13). They used CuAAC (copper-catalyzed 1,3-dipolar azide-alkyne cycloaddition) reactions to obtain new mono (C-28) and disubstituted (C-3 and C-28) derivatives in which the sugar unit is connected to the betulin system through various linkers, ester or eter, containing a 1,2,3-triazole ring. The structure of the linker results from the structure of the elements that constitute it. In one series, the carbon–carbon triple bond came from an alkynyl derivative of betulin, and the other reagent was a sugar with an azide function (**14a–d** and **15a–d**). Another series was obtained by reacting an azide derivative of betulin with acetylated propargyl *O*-glucoside **16a,b**. Cytotoxic activity was determined both for derivatives in which the hydroxyl groups of sugar were acylated and for the unprotected ones.

The study of activity against the HCT-116 line showed that newly designed glycoconjugates inhibited the proliferation of cancer cells, which was weaker or comparable to betulin **1**. Only 3,28-disubstituted betulin with a linker containing a 1,2,3-triazole ring without a sugar unit **17** was characterized by higher activity but was also very toxic [77].

**Figure 14:**
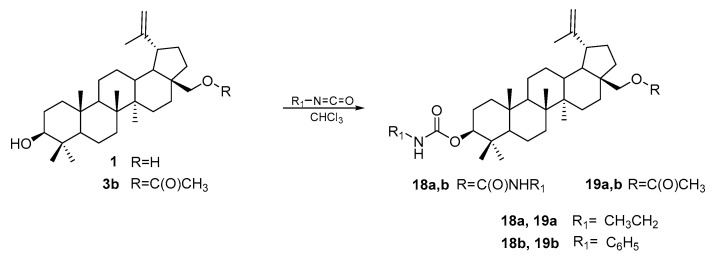
Synthesis of carbamate derivatives of betulin **18a,b** and 28-acetylbetulin **19a,b** [78].

The study of the cytotoxic activity of carbamate derivatives of BE showed that these compounds have higher activity against the DLD-1, HCT-8, HCT-116, HT-29, and SW480 lines compared to BA (IC_50_ is equal to 11.87; 13.10; 10.80; 13.93; and 6.48 μM, respectively). Compounds **18a,b** and **19a,b** were synthesized by the team of Kommera et al. in the reaction of betulin **1** and 28-acetylbetulin **3b** with ethyl and phenyl isocyanates in a chloroform solution at a temperature of 60 °C (Figure 14).

The most active compounds against the colon cancer cell line (SW480) in the tested series were the ethyl carbamate derivatives **18a** (IC_50_ = 2.77 μM) and **19a** (IC_50_ = 1.77 μM). These compounds were characterized by low activity in relation to normal cells (the SI is over 36 and equal to 13.76, respectively). Phenyl derivatives **18b** and **19b** showed no activity against the tested cancer lines [78].

**Figure 15:**
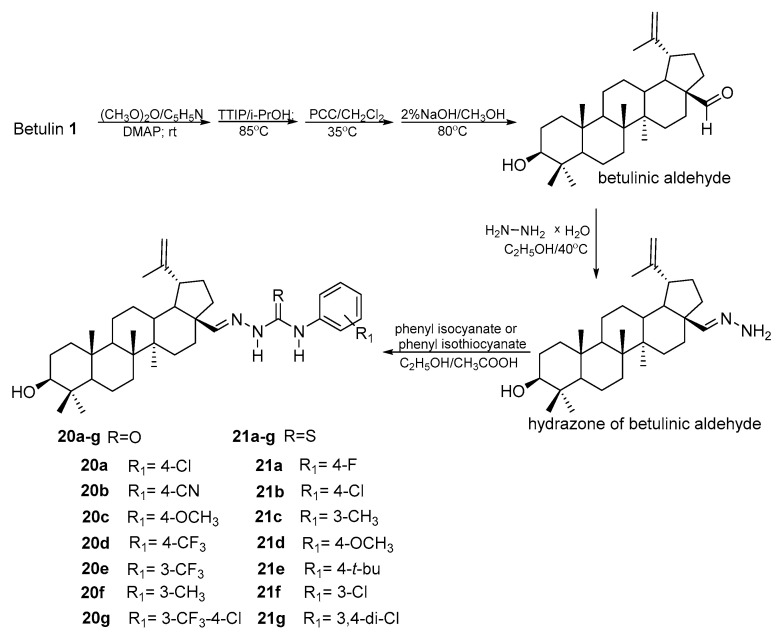
Synthesis of derivatives of betulin with semicarbazone moieties **20a–g** and thiosemicarbazone moieties **21a–g** [79].

The transformation of BE into betulinic aldehyde opened a new direction in the synthesis of derivatives whose structure resulted from the reactivity of the formyl group [79,80,81]. In the reaction of betulinic aldehyde with hydrazine hydrate, carried out in an ethanol solution at a temperature of 40 °C, betulin hydrazone is formed. This compound was reacted with phenylisocyanates and phenylthioisocyanates containing various substituents in the aromatic ring. In this way, two series of betulin derivatives were obtained—semicarbazones (**20a–g**) and thiosemicarbazones (**21a–g**) (Figure 15) [79]. 

The newly synthesized compounds were tested on the CRC cell line (HCT-116) and also against HCC cells (HepG2). Among the tested derivatives, the highest activity, comparable to the reference drug (mitomycin, IC_50_ = 11.36 μM) and higher than that of betulin (IC_50_ = 30.02 μM), towards HCT-116 cells was determined for **20g** (IC_50_ = 11.36 μM), which was also characterized by selectivity of action when tested on normal gastric cells (GES-1; IC_50_ = 144.63 μM). The synthesized compounds performed better against the HepG2 line. Derivatives **20a**, **21b**, and **21f** showed higher activity (IC_50_ = 8.93; 8.40; and 6.87 μM, respectively) than mitomycin (IC_50_ = 27.12 μM) and betulin (IC_50_ = 20.47 μM), which were also characterized by low toxicity (for GES-1 is equal to 196.18; 203.32; and 214.60 μM, respectively). 

**Figure 16:**
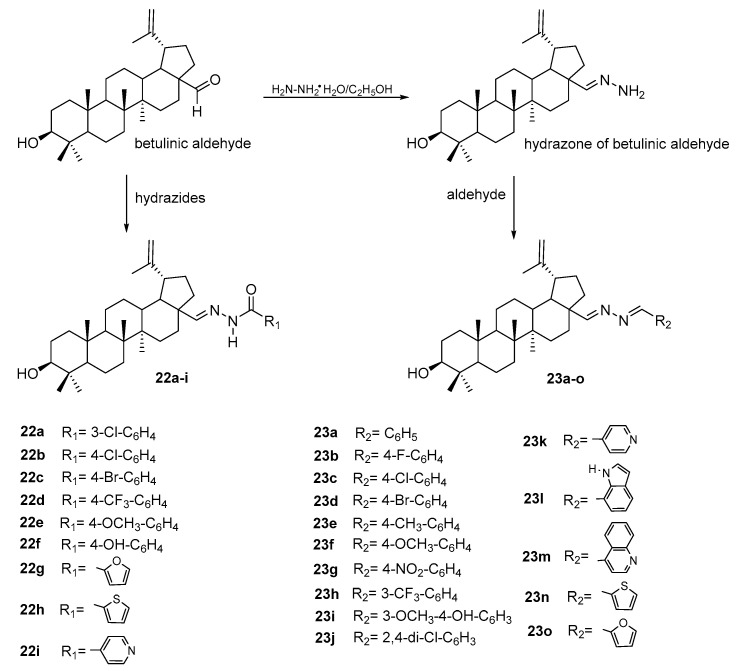
Structure of derivatives of betulin with hydrazide–hydrazone moieties **22a–i** [80] and **23a–o** [81].

The reactions of the aldehyde group with amine derivatives were also used in the synthesis of two further series of derivatives (Figure 16). In the first case, betulinic aldehyde reacted with hydrazides of various structures, obtaining derivatives containing hydrazide-hydrazone moieties **22a–i** [80]. Another series of compounds, **23a–o**, was created in the reaction of betulinic aldehyde hydrazone with aldehydes [81]. 

Studies of the cytotoxic effect on HCT-116 cells conducted for this group of derivatives showed that the hydrazide and hydrazine derivatives containing a 4-trifluoromethylphenyl substituent (**22d** and **23h**) were the most active (IC_50_ equal to 13.70 and 12.55 μM). Derivatives containing the pyridine ring **22i** (IC_50_ = 9.30 μM) and **23k** (IC_50_ = 9.32 μM) had the strongest effects on human hepatocellular carcinoma cells (HepG2) [79,80,81].

**Figure 17:**
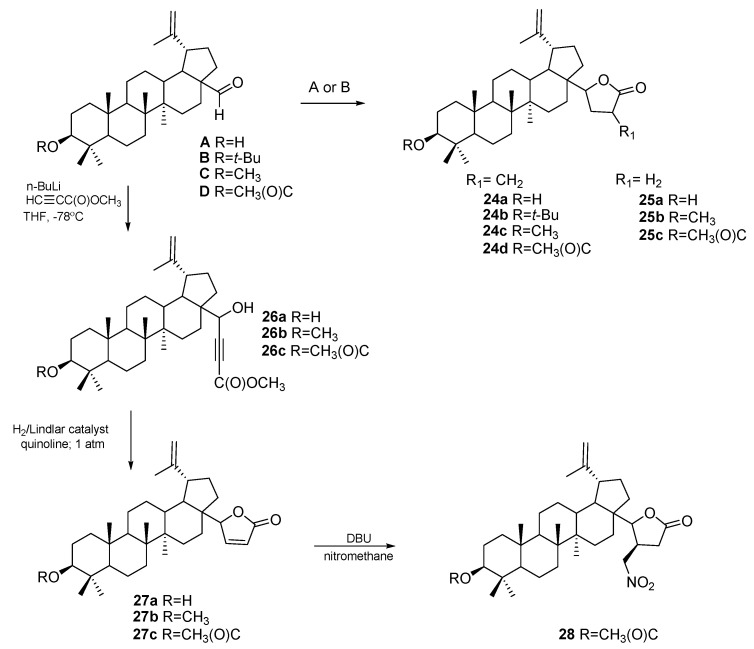
Synthesis of betulin derivatives **24a–d**, **25a–c**, **26a–c**, **27a–c**, and **28**. Procedure A (for **24a–d**): methyl 2-(bromomethyl)acrylate, Zn, THF, and ultrasound; Procedure B (for **25a–c**): samarium, 1,2-diiodoethane, THF, ultrasound, and next methyl acrylate, *tert*-butanol, THF, 0 °C [82].

Betulinic aldehyde **A** and its 3-substituted ether **B**, C, and ester derivatives **D** were also used as an intermediate for the synthesis of other types of BE derivatives (Figure 17). In the reaction of compounds **A–D** with methyl 2-(bromomethyl)-acrylate catalyzed via ultrasound, butyrolactones **24a–d** were formed in the tetrahydrofurane (THF) solution in the presence of zinc (Dreiding–Schmidt method). Butyrolactones **25a–c** were obtained from derivatives **A**, **C**, and **D** in a two-step synthesis. First, a sonicated mixture of samarium, 1,2-diiodoethane, and tetrahydrofuran was prepared, and then reductive coupling was performed by adding the appropriate aldehyde derivatives with methyl acrylate. 

Compounds **A**, **C**, and **D** with a formyl group were also reacted with methyl propiolate in tetrahydrofuran in the presence of *n*-BuLi to obtain 3-substituted methyl propiolates **26a–c**. These compounds were reacted with hydrogen in the presence of a Lindlar catalyst in ethyl acetate, transforming them into butenolides **27a–c**. Additionally, butenolide **27c** was transformed into compound **28** in the nitromethane addition reaction (Michael addition in the presence of DBU:1,8-Diazabicyclo[5.4.0]undec-7-ene) [82]. 

**Figure 18:**
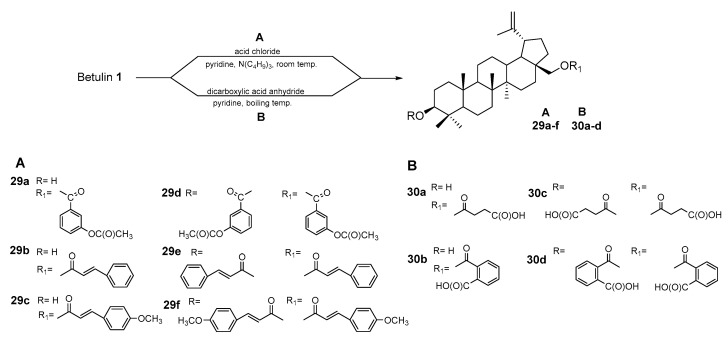
Scheme for the synthesis of betulin esters **29a–f** and **30a–d** (different procedures are indicated as A or B) [83].

The highest activity in relation to the tested cell lines (DLD-1, HT-29, HCT-8, HCT-116, SW480) was determined for propiolates **26a–c** [82]. In addition to semicarbazones, hyrdazines, and hydrazones, ester and carboxyacyl derivatives of betulin have also been investigated as agents against HCC. Flekhter et al. [83] described the conversion of BE to mono and diester derivatives by reaction with chlorides of such carboxylic acids as 3-acetoxy benzoic, cinnamic, and 4-methoxycinnamic (Figure 18, pathway A). As the second type of acylating agent, they used dicarboxylic acid anhydrides, succinyl, and orthophthalic (Figure 18, pathway B).

The obtained ester derivatives were tested for hepatoprotective effects on rat livers in models of experimental hepatitis caused by agents such as carbon tetrachloride, tetracyclines, and ethyl alcohol. Ester **30d** (betulin bishemiphtalate) reduced toxicity and restored hepatocyte function. The hepatoprotective effect of betulin derivatives, therefore, may be of importance in the prevention of HCC development, mainly caused by harmful factors such as alcohol.

**Figure 19:**
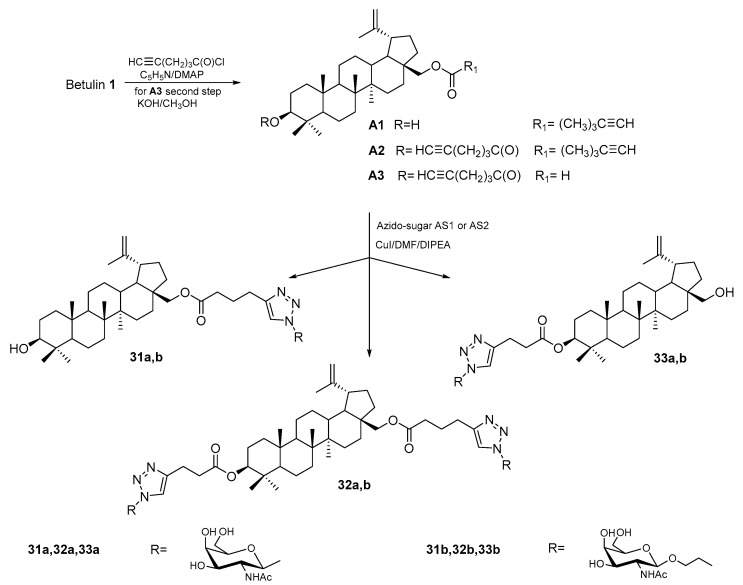
Scheme for the synthesis of betulin glycoconjugates **31a–33a** and **31b–33b** [84].

To design active compounds in HCC, Yamansarov et al. [84] used the reaction with hexynoic acid chloride and converted the obtained two monoesters (**A1** in the C-28 position and **A3** in the C-3 position) and the diester (**A2** in C-3 and C-28) of BE into the appropriate glycoconjugates of betulin and N-acetyl-D-galactosamine (GalNAc) **31a–33a** and **31b–33b** (Figure 19).

Assessment of the effect of the conjugates on two HCC cell lines, HepG2 and Huh7, showed that compounds **32a** (IC_50_ > 50 μM) and **32b** (IC_50_ equal to 25.9 and 47.2 μM) have a weaker effect than betulin **1** (IC_50_ equal to 4.2 and 5.8 μM).

**Figure 20:**
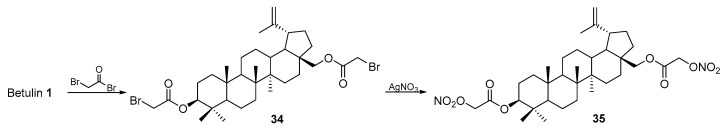
Scheme for the synthesis of 3,28-di-(2-nitroxy-acetyl)-oxybetulin **35** [85].

Another derivative containing ester groups in positions C-3 and C-28 was obtained by reacting betulin **1** with bromoacetyl bromide [85] (Figure 20). The presence of terminal bromine atoms in product **34** enabled the reaction with silver nitrate, which, in the final stage, gave 3,28-di-(2-nitroxy-acetyl)-oxybetulin **14**.

The authors investigated the anticancer activity and cytotoxic mechanisms of compound **35** against Huh7 cells [85].

**Figure 21:**
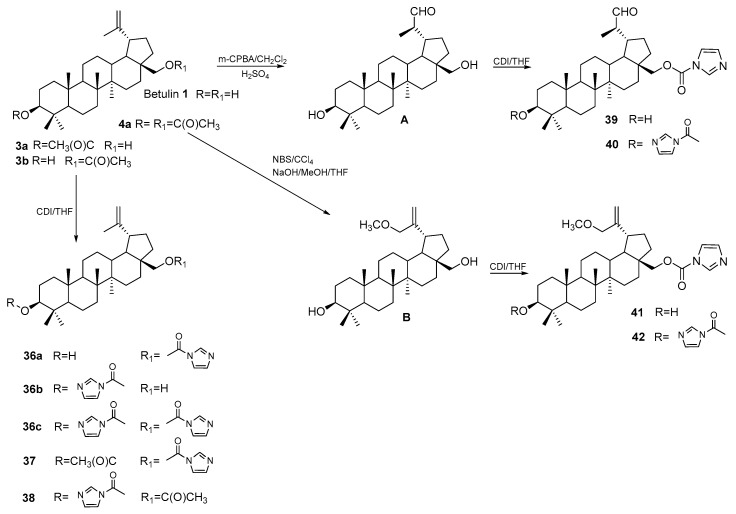
Scheme for the synthesis of carbamate derivatives of betulin **36a–c** and **37–42** [86].

In the search for new structures of potential drugs acting against liver cancer, carbamate derivatives of betulin **1** and betulin **A** 20*R*-aldehyde and 30-methoxybetulin **B** were synthesized. Carbamate derivatives were obtained in the reaction with 1,1′-carbonyldiimidazole (CDI) in a tetrahydrofuran solution. The reactions were carried out at reflux temperature under a nitrogen atmosphere. Among betulin derivatives (Figure 21) **36a–c** and **37–42** tested on HepG2 cells, monosubstituted compounds **36a**, **36b**, and **41** showed improved cytotoxic activity (IC_50_ equal to 4.20, 2.00, and 8.3 μM, respectively) compared to BA (IC_50_ = 36.4 μM) [86].

**Figure 22:**
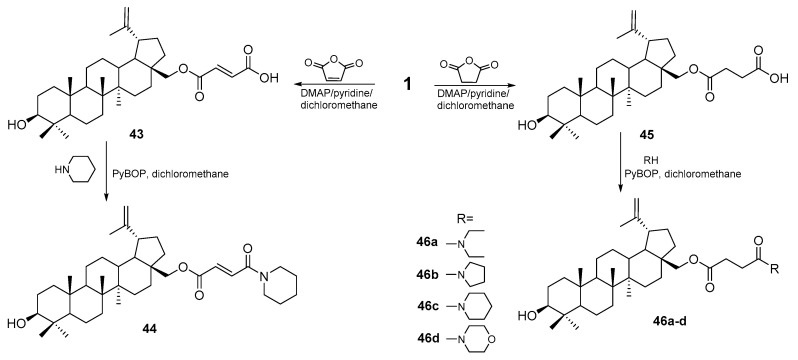
Scheme for the synthesis of betulin derivatives **43–45** and **46a–d** [87].

The reaction of betulin with succinyl or maleic anhydride and then the transformation of the free carboxyl group into the amide function in the reaction with tertiary amines of various structures carried out in dichloromethane in the presence of PyBOP (benzotriazol-1-yloxytripyrrolidinophosphonium hexafluorophosphate) allowed obtaining a series of derivatives **41–45** and **46a–d** (Figure 22), which were tested against MGC-803 cells (human GC cell line). The highest activity was determined for compounds **44** and **46c** (piperidine derivatives), which showed the greatest activity (IC_50_ = 5.3 and 4.3 μM). Compound **46c** was also characterized by high selectivity of action (SI = 8.23) compared to the mouse embryo fibroblast cell line [87]. 

**Figure 23:**
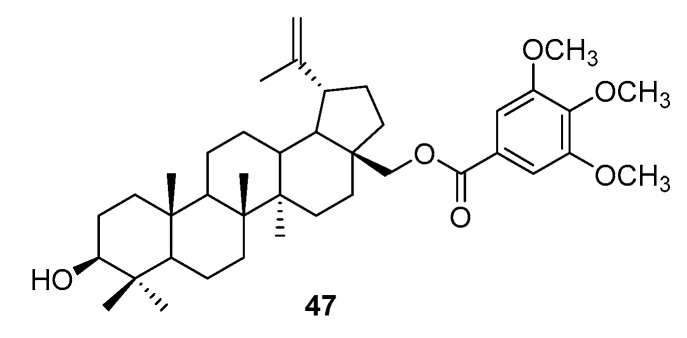
Structure of betulin derivative **47** [88].

In relation to the human GC cell line BGC 823, the monoester (at C-38) of betulin **1** and 3,4,5-methoxybenzoic acid was tested. The synthesis of compound **47** was performed under standard conditions (DCC/DMAP) (Figure 23). The obtained product did not show any cytotoxic activity towards the tested cells [88]. 

## 4. Effects of Betulin and Its Derivatives on Gastrointestinal Tract-Related Cancers

### 4.1. Colorectal Cancer

CRC is one of the most frequent types of gastrointestinal cancer, making it the third leading cause of mortality among people worldwide [89,90]. Risk factors, i.e., physical inactivity, poor diet, and smoking, are crucial in its development [89,90]. Only a small percentage of patients are associated with genetic predispositions [89].

Current therapies are selected according to the stage of disease progression, which is directly related to the division of colorectal cancer into resectable or unresectable [90,91,92]. In the first case, the approach of first choice is to remove a fragment of the neoplastic lesion with a margin of healthy tissue. However, sometimes this method, especially in higher stages associated with metastatic CRC (mCRC) causing liver or lung metastases, is not enough, so additional combination therapy with radiotherapy, chemotherapy, or targeted therapy is used [90,91,92].

In the case of unresectable cancer, chemotherapy is used until removal of the lesion is possible, for which purpose the main cytostatic drugs administered are 5-FU, irinotecan, oxaliplatin, capecitabine, or their combination, e.g., 5-FU plus folinic acid—an agent that increases the activity of 5-FU in preventing disease recurrence [90,91,92]. The use of such therapies, however, also has adverse effects on the patient’s normal cells, causing many side effects or inducing systemic toxicity. Therefore, with this in mind, new medicinal substances with antitumor properties that are selective only towards cancer cells are being studied. One of them is betulin and its derivatives, which have improved pharmacological and pharmacokinetic properties that also increase their bioavailability [93].

In a study conducted by Grymel et al. [73], it was reported that the obtained triphenylphosphonium derivatives of BE, i.e., 3-*O*-Acetyl-28-*O*′-(3′-(3″-triphenylphosphoniopropyloxycarbonyl)propanoyl)betulin bromide **8a** and 3-*O*-Acetyl-28-*O*′-(3′-(4″-triphenylphosphoniobutylxycarbonyl)propanoyl)betulin bromide **8b**, were found to increase the cytotoxic effect on CRC cells of the HCT-116 line compared to betulin itself [73]. In addition, it has been shown that the new conjugates obtained in this way lead to enhanced transport of the compound through the mitochondrial membrane [73]. Interestingly, the introduction of a triphenylphosphonium group into betulin’s backbone structure also showed a two-fold reduction in toxicity against normal cells of the NHDF line, indicating that the resulting derivatives have a targeted effect on cancer cells themselves and may provide a starting point for the development of new drug formulations [73].

In contrast, in an experiment conducted by Wang et al. [79], betulin derivatives containing a thio/-semicabazone group showed low cytotoxic activity against colon cancer cells of the HCT-116 line. Interestingly, only the obtained derivative lup-20(29)-ene-3β-ol-28-N-((3-trifluoromethyl-4-chloro)phenyl)semicarbazide **20g** displayed significant cytotoxic activity against the CRC line [79]. This may indicate that the introduction of the thiosemicarbazide grouping has a minor effect on colorectal cancer, which may be due to a different expression profile of receptors on the cell surface or impaired transport across the cell membrane [79].

In some of the studies conducted to date on CRC cell lines on the effects of betulin derivatives, an SI was also determined, the value of which varied significantly between cell lines as well as between derivatives. Due to the fact that SI values were not determined for most of the betulin derivatives presented in the review and no studies were conducted on normal cell lines, only those SI values for betulin derivatives that were evaluated are presented. For the derivative **4c=3b** for the HT-29 cell line, the SI value was 0.57 ± 0.1, indicating poor selectivity of this derivative [69]. Similar results were obtained for derivative **13b** tested on the DLD-1 cell line, where the SI value was 0.68 ± 0.1 [76].

However, derivatives **18a** and **19a** showed high selectivity towards colorectal cancer cells with low toxicity towards normal fibroblast cells. SI values for compound **18a** were for the DLD-1 (>12.02), HCT-8 (>25.58), HCT-116 (>26.04), HT-29 (>16.13), and SW480 (>36.10) lines, accordingly. In contrast, for derivative **19a**, the SI values for the same cell lines were 3.52; 6.20; 5.67; 4.80; and 13.76, respectively [78]. 

Thus, depending on the group introduced, the therapeutic effect may be different and highly dependent on the type of cancer [73,79]. Some examples of other studies of the effects of BE and its derivatives on colorectal cancer cells are shown in Table 1.

**Figure 24:**
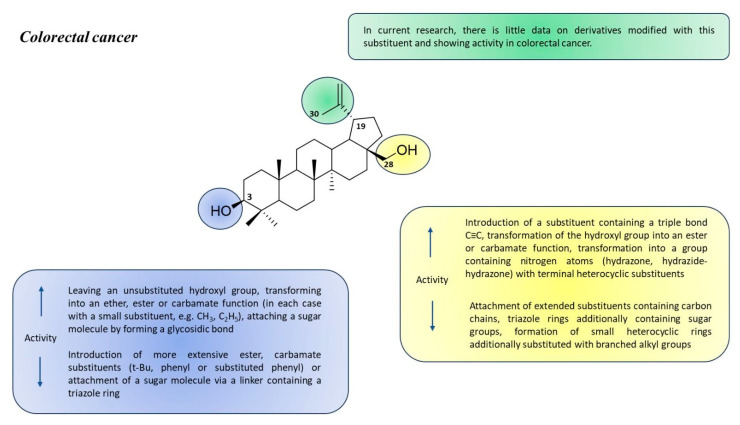
Structure–activity relationship for betulin derivatives effective against colorectal cancer.

According to the literature data presented above, betulin derivatives that have been synthesized as substances with potential activity against colorectal cancer are characterized by great structural heterogeneity. Based on the results of in vitro studies on various cell lines (HT-29, DLD-1, HCT-8, HCT-116, and SW480), it is possible to select the types of chemical modifications that may have a beneficial effect on enhancing the cytotoxicity of the obtained derivatives (Figure 24).

Among the compounds described, **26a–c** derivatives seem to be the most promising due to their high activity in the five tested cell lines. Determining their usefulness as potential medicinal substances requires further research.

### 4.2. Hepatocellular Carcinoma

HCC is currently classified as the fourth most lethal among all types of neoplasms [95,96]. The incidence and development of HCC often depend on lifestyle, where consumption of large amounts of alcohol, which can lead to cirrhosis, is crucial [95,96]. Undoubtedly, infection with the hepatitis B and C viruses is also essential, which can affect the morbidity rate and symptoms in the early stages [95,96]. The prognosis of HCC is poor, however, treatment options, mainly based on immunotherapy, are available [95,97].

The method of treatment mainly depends on the stage of the disease, and in the early stages, surgical resection or liver transplantation is used [97]. Patients with highly progressive cancer are treated with immunotherapy using sorafenib, an inhibitor of multiple kinases, including Raf, c-kit, and VEGF receptors [97]. For HCC, standard chemotherapies are not effective [97].

Due to the relatively limited possibility of therapy, researchers are looking for new solutions in the field of HCC pharmacology, where attention is focused on substances of natural origin, i.e., betulin. In a study conducted by Soliman et al. [98] on cell lines including the HCC line HepG2, they showed that a betulin derivative, 13-dehydrobetulin, isolated from the stem of the *Ziziphus* plant grown in a hotter climate, results in enhanced cytotoxicity against HCC compared to the native form of betulin [98]. The findings of the study, consequently, may contribute to the exploration of more plants cultured in other environmental conditions with regard to the search for new medicinal substances in the pharmacotherapy of HCC.

The team of Liu et al. [99], in contrast, studied BA on HCC cancer lines SMMC-7721 and HepG2, where they demonstrated an inhibitory effect of this compound on the proliferation of cancer cells [99]. Moreover, they found that this effect is mediated via a suppressive effect on the PI3K/AKT/mTOR signaling pathway, which stimulates autophagy, leading to cell death [99]. Such studies have revealed the significant impact of betulin and its hydrocarbon derivatives on new therapeutic possibilities for HCC [99]. 

In addition, it may be suggested that betulin itself has a low cytotoxic effect against hepatocellular carcinoma cell lines; however, a relatively high cytotoxic effect was observed with carbamate derivatives. Therefore, this suggests that, in the future, derivatives with such a grouping may prove effective against HCC [86].

However, there are still a relatively small number of studies on their effects on HCC, yet several studies on the assessment of the impact of betulin and its derivatives on HCC cells are presented in Table 2.

### 4.3. Gastric Cancer

GC is classified as the fifth most common cancer incidence, mostly affecting men from highly endemic countries such as Asia [101,102,103]. Some of the factors causing the disease include chronic gastritis, which leads to neoplastic transformation [24]. *Helicobacter pylori* infection is also one of the frequently mentioned risk factors for GC, which is directly related to the aforementioned chronic inflammation [102,103]. Among other contributing factors are the usual poor diet, high salt, smoking, obesity, and reflux disease. Since GC is highly heterogeneous, it is difficult to treat [101,103]. It is assumed that in the case of metastatic GC, standard chemotherapy is used. In other cases, the method of first choice is either surgical resection or combination therapies [103]. Increasingly, targeted therapy is also being used, with trastuzumab, a monoclonal antibody directed against human epidermal growth factor receptor type 2 (HER2), leading the way [103].

Due to the highly variable profile of GCs among patients, the challenge of designing new therapies is a major obstacle [101,102,103]. Compounds of natural origin, due to their proven antitumor properties, may prove to be a breakthrough in the development of new drugs. The antitumor, anti-inflammatory, and antiviral features are exhibited by betulin; therefore, it seems to be a suitable starting point for novel GC treatments.

Research by the team of Li et al. [104] performed on the SGC7901 gastric cancer line exposed to betulin proved that the compound promotes apoptosis in the neoplastic cells via a mitochondria-related pathway [104]. In addition, betulin generates large amounts of reactive oxygen species, resulting in a decrease in anti-apoptotic proteins, i.e., Bcl-2. This causes the translocation of Bax and Bak proteins and the release of cytochrome c and Smac [104]. Additionally, the team came up with the conclusion that such an action can also lead to the sensitization of GC cells to apoptosis-associated signals [104].

A similar study was conducted by Chen et al. [105] on three gastric cancer lines (BCG-823, MNK45, and 293T) treated with betulinic acid [105]. Using molecular techniques, they showed that the test compound inhibits the proliferation and migration of GC cells by increasing the expression of vasodilator-stimulated phosphoprotein (VASP) and inhibiting NF-kB activity [105]. These results show that the hydroxyl derivative of betulin is of great value in the development of new compounds with potential antitumor activity targeting genes responsible for cytoskeleton rearrangement required for metastasis [105].

### 4.4. Esophageal Cancer

EC is one of the less common gastrointestinal cancers but is ranked ninth in terms of morbidity [106,107]. In most cases, it is diagnosed at the metastatic stage, resulting in a relatively poor prognosis with an overall 5-year survival rate of less than 20% [106,107]. Standard risk factors include alcohol consumption, smoking of tobacco products, and consumption of hot beverages [106]. It is also assumed that a higher incidence of EC is observed in people with reflux disease or bacterial infections, mainly *Helicobacter pylori* [106]. Treatment of EC is relatively poor and consists of surgical resection, chemotherapy, liquid nitrogen spray cryotherapy, or a combination of these methods [106,107]. In cases of advanced disease, only palliative treatment is used [106].

Due to the limited therapeutic options for EC, new substances are being sought as an alternative for EC patients. In a study conducted by Chen et al. [108] on EC cells of the TE-11 line, they showed that the use of BA increases the sensitivity of the cancer cells to the standard chemotherapeutic agent, cisplatin [108]. They demonstrated that the compound acts by inhibiting proliferation and decreasing their specificity, as well as inducing cell death via pyroptosis [108]. Therefore, this information provides new avenues for betulinic acid research in terms of using BA to support the treatment of EC with chemotherapeutic agents. In addition, in order to improve the bioavailability of betulin derivatives, it may be useful to develop new formulations based on nanoparticles or other carriers, i.e., viruses and virus-like particles, to further increase the bioavailability and targeting of a specific cell type [109].

Examples of other studies of the effects of betulin and its derivatives on gastric and esophageal cancer cells are presented in Table 3.

### 4.5. Pharmacokinetic Profile and Animal Studies of Betulin and Its Derivatives in Gastrointestinal Cancers

Undoubtedly, BE and its new synthetic derivatives are one of the most widely developed topics currently in terms of new drugs with potential antitumor activity. Although numerous studies are already being carried out on betulin itself, mainly in the antiviral and antibacterial aspects, its pharmacokinetic profile is not fully understood due to its physicochemical properties (i.e., poor water solubility, lipophilicity, poor bioavailability, etc.) [35]. However, an attempt to study the pharmacokinetics of BE was made by Jäger et al. [113] using blood samples collected from Beagle dogs and Sprague Dawley rats as animal models. In dogs, betulin was administered subcutaneously (s.c.) at a dose of 30, 100, and 300 mg/kg over 4 h, while in rats, it was administered intraperitoneally (i.p.) at a dose of 60, 180, and 540 mg/kg. Blood samples were also collected after 28 days. BE showed a time-dependent relationship over a 4 h period after administration, with a dose-independence level of 0.13 µg/mL in the serum of the test faders [113]. A similar effect was observed with subcutaneous administration in dogs, where the serum BE concentration after 28 days at a dose of 300 mg/kg was 0.13 µg/mL. In addition, the study did not find subchronic toxicity in any of the study models and concluded that the administration of betulin is safe [113].

In another study by the team of Pozharitskaya et al. [114], they examined the pharmacokinetic profile of betulin and nanoformulation (BN) obtained using the solvent exchange technique. The in vivo model consisted of rats that received endotracheally either BE or BN at a dose of 25.2 mg/kg. Blood samples were taken at intervals of 15, 30 min, and 1.2 and 4 h after administration of the substance [114]. They showed that the maximum plasma concentration of BN occurred 15 min after administration and was 15.5 μg/mL, while for BE, it was 6.9 μg/mL after 1 h of administration. Interestingly, the lowest concentrations were found in the heart, while high values were also observed in the lungs and liver [114]. This suggests that the pharmacokinetic profile is dependent on the formulation of betulin used as well as the route of administration and dose.

Currently, there are no data available on the pharmacokinetic profiles of the derivatives discussed in the review, and no clinical trials of betulin derivatives are being conducted due to poor evidence from the animal model. However, most of the information appearing in clinical trials relates to betulinic acid derivatives, which also show promising anticancer activity. Therefore, betulin derivatives themselves are a promising new potential source of anticancer agents in gastrointestinal cancers, but further research is still required, mainly in the areas of pharmacokinetics, drug formulation, and in vivo studies.

## 5. Conclusions

This review presents the most recent reports to date on the synthesis, pharmacological effects, and molecular mechanisms of action of betulin and its derivatives in gastrointestinal-related cancers. Depending on the substituents in the structure of betulin, different effects can be obtained, both therapeutic and those related to specific effects on a particular cell type. The cytotoxic effect of the derivatives depends on the cell lines, which may be associated with genetic and epigenetic differences in each line, possibly resulting in a varying mechanism of action. The derivatives listed in the tables show the highest therapeutic potential due to the fact that, in most cases, the IC_50_ of a certain derivative is greater than that of the reference compound. Therefore, this makes it possible to study them further as well as to try new formulations of these compounds. However, more research on the in vivo model is needed to evaluate its applicability for clinical trials.

## Figures and Tables

**Table 1:** Examples of other studies of the effects of betulin and its derivatives on colorectal cancer cells.

Derivative	CRC Cell Lines	Effects	IC_50_	Ref.
**1**	DLD-1	Leads to a change in the morphology of CRC cells and affects the induction of apoptosis and reduction in motility	6.6 μM	[75,76]
	HCT-8		n/d	n/d
	HCT-116		30.02 ± 1.22 μM 27.46 μM 25.7 ± 2.07 μM	[79,80,81]
	HT-29		4.3 μM	[94]
	SW480		n/d	n/d
**3d** (Figure 7)	HT-29	Hight cytotoxic effect against CRC line than BA (IC_50_ = 84.5 ± 6.6 μM); the obtained derivatives show higher polarity	13.6 ± 5.7 μM	[68]
**4c=3b** (Figure 7)	HT-29	The compound 28-*O*-chloroacetylbetulin shows greater selectivity towards specific neoplastic cells; induces apoptosis in HT-29 cell line; and compound **4c=3b** exhibits higher cytotoxic effect than BA (IC_50_ = 13.93 ± 0.46 μM)	10.96 ± 0.87 μM	[69]
**4d** (Figure 7)			27.63 ± 1.88 μM	
**4c=3b** (Figure 7)	DLD-1, HCT-8, HCT-116, HT-29, SW480	α-monomer of d-glucopyranoside induces apoptosis in neoplastic cells through activation of caspase 2 and 8. Cytotoxicity similar to the BA (IC_50_ = 11.87 ± 0.29 μM; 13.10 ± 0.50 μM; 10.80 ± 0.24 μM; 13.93 ± 0.46 μM; and 6.48 ± 0.12 μM, respectively), although the derivatives exhibit improved properties	DLD-1—13.12 ± 1.05 μM HCT-8—17.98 ± 0.44 μM HCT-116—10.71 ± 0.65 μM HT-29—10.96 ± 0.87 μM SW480—13.68 ± 0.39 μM	[74]
**10a** (Figure 11)			DLD-1—4.45 ± 0.23 μM HCT-8—10.19 ± 0.03 μM HCT-116—7.46 ± 1.43 μM HT-29—9.80 ± 0.05 μM SW480—9.52 ± 0.04 μM	
**18a** (Figure 14)	DLD-1, HCT-8, HCT-116, HT-29, SW480	Higher cytotoxicity against colon cancer cells than the effect of BA (IC_50_ = 11.87 μM; 13.10 μM; 10.80 μM; 13.93 μM; and 6.48 μM, respectively)	DLD-1—8.32 μM HCT-8—3.91 μM HCT-116—3.84 μM HT-29—6.20 μM SW480—2.77 μM	[78]
**19a** (Figure 14)			DLD-1—6.93 μM HCT-8—3.99 μM HCT-116—4.30 μM HT-29—5.07 μM SW480—1.77 μM	
**13b** (Figure 12)	DLD-1	Cytotoxic activity towards neovascular cells; exhibits higher cytotoxic effect than BE (IC_50_ = 6.6 ± 0.3 μM) and BA (IC_50_ = 10.3 ± 0.4 μM); and high polarity of the obtained compounds allows their formulation via injection for in vivo studies	1.9 ± 0.9 μM	[76]
**22f** (Figure 16)	HCT-116	Stronger anticancer activity compared to BE (IC_50_ = 25.7 ± 2.03 μM)	16.7 ± 1.14 μM	[80]
**22i** (Figure 16)			18.0 ± 1.14 μM	
**22d** (Figure 16)			13.7 ± 0.077 μM	
**26a** (Figure 17)	DLD-1, HCT-8, HCT-116, HT-29, SW480	Higher cytotoxic effect than BA (IC_50_ = 17.5 μM; 17.8 μM; 13.3 μM; 16.1 μM; and 6.4 μM respectively); the strength of the effect depends on the cell line	DLD-1—6.0 μM HCT-8—2.6 μM HCT-116—4.8 μM HT-29—4.9 μM SW480—5.9 μM	[82]
**26b** (Figure 17)			DLD-1—3.6 μM HCT-8—6.0 μM HCT-116—3.5 μM HT-29—5.0 μM SW480—3.6 μM	
**26c** (Figure 17)			DLD-1—5.7 μM HCT-8—8.2 μM HCT-116—3.5 μM HT-29—4.7 μM SW480—5.5 μM	

CRC—colorectal cancer; BE—betulin; BA—betulinic acid; and n/d—no data.

**Table 2:** Examples of other studies of the effects of betulin and its derivatives on hepatocellular carcinoma cells.

Derivative	HCC Cell Lines/Animal	Effects	IC_50_	Ref.
**1**	HepG2; SK-HEP-1	HepG2 cells are more sensitive to betulin than SK-HEP-1 cells, due to lower expression of caspase-9 in the SK-HEP-1 line; higher expression of caspase-9 results in cell resistance to betulin; and betulin triggers apoptosis through a mitochondrial pathway related to caspase-9 and through the release of cytochrome c and Smac	HepG2—10.1 ± 1.38 μM; SK-HEP-1—58.5 ± 3.00 μM	[100]
**32a** (Figure 19)	HepG2, Huh7/BALB/c mice	Investigated conjugates of betulin derivatives show high selectivity against HCC cells in both in vitro and in vivo models; exhibit a high cytotoxic effect by generating reactive oxygen species	>50 μM for all cancer lines	[84]
**32b** (Figure 19)			HepG2—25.9 μM; Huh7—47.2 μM	
**35** (Figure 20)	Huh7	The obtained betulin derivative downregulates the expression of Bcl-2 protein resulting in loss of mitochondrial membrane potential and release of cytochrome c; arrests the cell cycle in the G2/M phase and induces cell apoptosis	13.1 ± 1.37 μM	[85]
**36a** (Figure 21)	HepG2	Higher cytotoxicity than BE (IC_50_ = 36.4 ± 1.5 μM) and BA (IC_50_ = 4.2 ± 0.3 μM)	4.20 ± 0.03 μM	[86]
**36b** (Figure 21)			2.00 ± 0.4 μM	
**41** (Figure 21)			8.3 ± 0.4 μM	
**20a** (Figure 15)	HepG2	Higher cytotoxicity than BE (IC_50_ = 20.42 ± 1.22 μM)	11.64 ± 0.62 μM	[79]
**21b** (Figure 15)			8.40 ± 0.34 μM	
**21f** (Figure 15)			6.87 ± 0.76 μM	
**22i** (Figure 16)	HepG2	Higher cytotoxic effect on HCC cells than BE (IC_50_ = 21.0 ± 0.72 μM)	9.3 μM	[81] [80]
**23k** (Figure 16)			9.32 μM	

HCC—hepatocellular carcinoma; BE—betulin; BA—betulinic acid; and n/d—no data.

**Table 3:** Examples of other studies of the effects of betulin and its derivatives on gastric and esophageal cancer cells.

Derivative	Cell Lines/Animal	Effects	IC_50_	Cancer	Ref.
**1**	EPG85-257P	Strong cytotoxicity of compounds against drug-resistant cell lines; betulinic acid shows a higher cytotoxic effect than betulin	18.74 μM	GC	[110]
**2**			6.16 μM		
**46c** (Figure 22)	MGC-803	The betulin derivative shows a high cytotoxic effect against gastric cancer line; in addition, induces morphological changes and inhibits proliferation by inducing apoptosis	4.3 ± 0.4 μM	GC	[87]
**1**	SGC7901	Inhibition of tumor cell proliferation; influence on pathways related to mitochondria and reactive oxygen species	5.75 μM	GC	[104]
**1**	BGC823	Betulin shows weaker cytotoxic effect against BGC823 lines than betulinic acid (IC_50_ = 46.26 ± 3.75 μM)	>100 μM	GC	[88]
**2**	NCI-N87; SNU-16/BALB/c mice	Compound inhibits proliferation and promotes apoptosis; inhibits the EMT pathway and, in a mouse model, delays tumor growth	n/d	GC	[111]
**2**	YES-2/SCID mice	Betulinic acid inhibits YES-2 cell line proliferation; betulinic acid induces an additive interaction together with irinotecan in a mouse model	YES-2—5.09 ± 1.10 μM	EC	[112]

GC—gastric cancer; n/d—no data; EMT—epithelial–mesenchymal transition; SCID—severe combined immunodeficiency; and EC—esophageal cancer.

## Data Availability

Available on request and with regulations.
